# Efficacy and safety of subcutaneous versus intravenous administration of PD-1/PD-L1 inhibitors in the treatment of solid tumors: a systematic review and meta-analysis

**DOI:** 10.3389/fonc.2026.1748414

**Published:** 2026-04-27

**Authors:** Peiye Wu, Zhanpeng Liang, Zhaoyang Wu, Enxi Zhao, Cantu Fang

**Affiliations:** Zhongshan Hospital of Traditional Chinese Medicine Affiliated to Guangzhou University of Traditional Chinese Medicine, Zhongshan, China

**Keywords:** intravenous administration, meta-analysis, PD-1/PD-l1 inhibitors, subcutaneous administration, systematic review

## Abstract

**Objective:**

The aim was to evaluate the differences in pharmacokinetics, clinical efficacy, and safety between subcutaneous (s.c.) and intravenous (i.v.) administration of PD-1/PD-L1 inhibitors in the treatment of solid tumors, and to explore the development history and future prospects of s.c. administration of PD-1/PD-L1 inhibitors.

**Methods:**

PubMed, Embase, and The Cochrane Library were searched for relevant studies from inception to October 10, 2025. Systematic reviews and meta-analyses were used to compare the pharmacokinetics, efficacy, and safety of s.c. and i.v. PD-1/PD-L1 inhibitors in the treatment of solid tumors. Review Manager 5.4.1 was used to analyze the data. Primary outcomes and endpoints included serum drug concentration、overall survival (OS), progression-free survival (PFS), objective response rate (ORR), and adverse events (AE).

**Results:**

This systematic review and meta-analysis included three randomized controlled trials with a total of 1243 patients (746 received s.c. administration, and 497 received i.v. administration). Compared to the i.v. administration, there were no significant differences in drug concentration, OS [HR = 0.86; 95% CI (0.68, 1.08); P = 0.19], PFS [HR = 1.06; 95% CI (0.92, 1.23); P = 0.42], or ORR [RR = 1.18; 95% CI (0.97, 1.43); P = 0.09] for the s.c. administration of PD-1/PD-L1 inhibitors. Regarding AE, arthralgia was more commonly associated with s.c. administration, whereas i.v. administration resulted in a higher incidence of injection site reactions.

**Conclusion:**

The pharmacokinetics of s.c.PD-1/PD-L1 inhibitors for the treatment of solid tumors are similar to those of i.v. administration, and no significant differences were found in efficacy and safety analyses; This finding supports s.c. administration as a viable and complementary alternative to traditional i.v. delivery.

**Systematic Review Registration:**

https://www.crd.york.ac.uk/prospero, identifier CRD420251161810.

## Introduction

The emergence of immune checkpoint inhibitors (ICIs) has brought profound changes to the field of cancer treatment, which is gradually developing from the traditional second or third line treatment to the first-line treatment option, and has become an important combination or alternative to chemotherapy or targeted therapy for a variety of cancers. PD-1/PD-L1 inhibitors are among the most commonly used ICIs in clinical practice. By binding to PD-1 or PD-L1, it blocks the interaction between PD-1 and its ligand PD-L1, thereby restoring the recognition and killing function of immune cells and preventing tumor cells from escaping immune surveillance (1).

Subcutaneous (s.c.) administration has significant advantages over intravenous (i.v.) administration. s.c. administration is easy to operate, basically does not damage blood vessels, can effectively reduce pain and anxiety of patients, and has the feasibility of self-administration, which is more suitable for the needs of long-term standardized management of cancer patients. Although s.c. anticancer agents have been used in clinical practice (2), PD-1/PD-L1 inhibitors approved by the U.S. Food and Drug Administration (FDA) and the European Medicines Agency (EMA) are still administered via the i.v. route (3–5). At present, the development of s.c. formulations of PD-1/PD-L1 inhibitors, as an alternative to traditional i.v., with preliminary data supporting its potential.

The urgency for optimizing cancer treatment strategies is underscored by the recent estimates from the Global Burden of Disease Study, which predicted that the incidence of cancer cases will reach 35 million cases per year by 2050, an increase of 77% from 2022, mainly driven by the demographic and epidemiological transition in the context of the aging population and changing risk factors in transitioning economies (6). The optimization of the more efficient, more scalable, and more patient-centric delivery models for immunotherapies, such as PD-1/PD-L1 inhibitors, becomes more than a question of convenience; it becomes a global health imperative.

We will summarize the phase III trials of s.c. versus i.v. PD-1/PD-L1 inhibitors, use systematic review and meta-analysis to evaluate the differences in pharmacokinetics, efficacy and safety between s.c. and i.v. in solid tumors, and explore the history and prospect of PD-1/PD-L1 inhibitor**s** s.c. administration.

## 2 Methods

This study was registered in the PROSPERO database (CRD420251161810) and conducted under the Preferred Reporting Project for Systematic Review and Meta-Analysis (PRISMA) statement. The purpose of this study was to compare the pharmacokinetics, efficacy and safety of s.c. and i.v. PD-1/PD-L1 inhibitors.

### 2.1 Search strategy

The following databases were searched for studies: PubMed, Embase, and the Cochrane library. The keywords or corresponding grid terms used to search the database were: ICIs, s.c., i.v., and others. The detailed search strategy is as described in the **Supplementary Material**. Search did not apply language or date restrictions. If overlapping data exists, the most complete and updated report is selected for inclusion in this meta-analysis. Manually review references from all eligible studies to find other relevant studies. The last search was dated October 10, 2025.

### 2.2 Eligibility criteria

Studies were screened independently by two authors. The following criteria were used to identify eligible studies: 1) Adult patients with solid malignant tumors confirmed by pathology and without previous anti-tumor immunotherapy, regardless of gender, regional race and tumor type; 2) Retrospective studies, cohort studies, case-control studies, cross-sectional studies and randomized controlled studies comparing the pharmacokinetics, efficacy and safety of s.c. and i.v. PD-1/PD-L1 inhibitors; 3) provide any of the following outcome indicators: serum drug concentration, overall survival (OS), progression-free survival (PFS), objective response rate (ORR), and adverse events (AE).

Exclusion criteria: (1) lack of PD-1/PD-L1 inhibitor i.v. control group; (2) studies that did not meet the above diagnostic criteria, failed to obtain the outcome indicators, or whose previous medical conditions may significantly affect the objectivity of the outcome indicators; And (3) studies presented as review articles, letters to the editor, animal studies, and case reports.

### 2.3 Study selection and data extraction

Two experienced investigators independently screened the retrieved literature, and any disagreements were resolved by consensus after consultation with a third investigator. Relevant articles were retrieved from literature databases. After removing duplicate articles through literature management software, the titles and abstracts of the remaining articles were scanned to further screen potentially eligible articles. Finally, the full text of these potentially eligible articles was obtained and read to identify the final included studies.

The extracted data included the sample size of the study population, baseline characteristics, interventions, and study outcomes. Two investigators extracted relevant data independently, and any discrepancies were resolved by consulting a third investigator. When multiple articles included overlapping patient populations, we preferentially extracted outcome data from the primary article with the largest sample size for early outcomes and the article with the longest follow-up for late outcomes.

### 2.4 Quality assessment of the literature

The quality of the literature was evaluated using the Cochrane bias risk assessment tool. The risk of bias was evaluated in terms of selection (random sequence generation and allocation concealment), implementation (blinding of investigators and subjects), measurement (blinded evaluation of study outcomes), follow-up (completeness of outcome data), reporting (selective reporting of study results) and others (other sources of bias).

### 1.5 Statistical methods

Review Manager 5.4.1 (Cochrane Collaboration software) was used to analyze the data, which were extracted from the articles or computed. OS, PFS, and AE are reported as hazard ratios (HR) and ORR as risk ratio (RR) with 95% confidence intervals. Heterogeneity between studies was assessed using the I^2^ test (level of test yield >50%). When P≥0.10 and I^2^ ≤ 50%, there was no significant heterogeneity among the studies, the fixed effect model was selected. If P<0.10 or I^2^>50%, heterogeneity between studies was considered significant, so a random effects model was used. P<0.05 was considered statistically significant.

## 3 Results

### 3.1 Literature screening process and key characteristics of included studies

**Figure 1:**
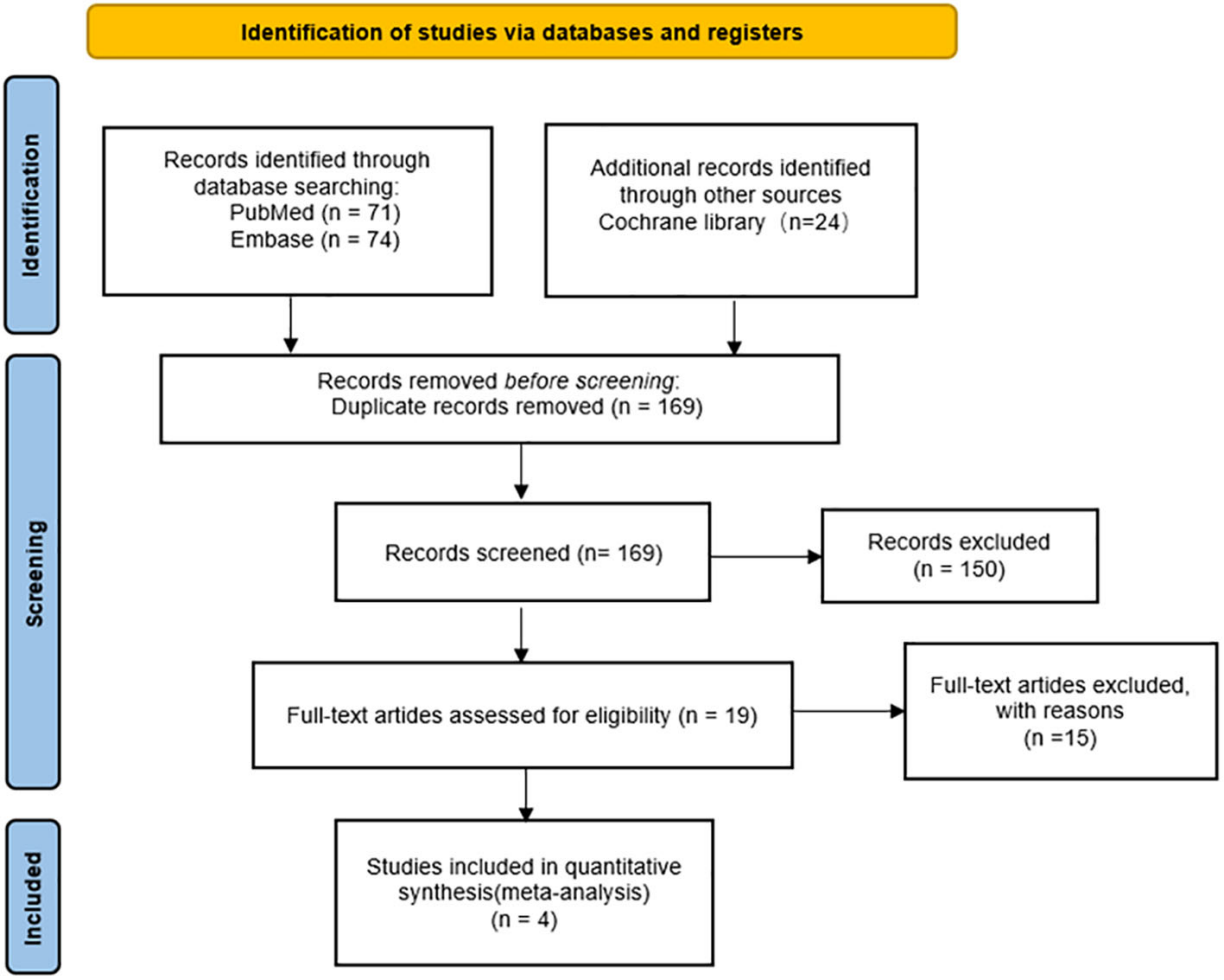
Study selection flowchart.

169 articles were obtained according to the preset search strategy. A total of 4 literatures met the requirements were screened by the title, abstract and full text reading. Four literatures reported three clinical trials (IMscin001 Part2 (7, 8), CheckMate67T (9) and 3475A-D77 (10)) respectively, and all of them were randomized controlled trials. Involving three drugs Atezolizumab, Nivolumab and Pembrolizumab, a total of 1243 patients were enrolled, including 746 in the s.c. group and 497 in the i.v. group. The screening process is shown in **Figure 1**, and the basic information of the included studies is shown in **Table 1**.

**Table 1:** Basic characteristics of included trials.

Study	Drug	Study population	Study design	Intervention	Sample size (Experimental/Control)
CheckMate 67T	Nivolumab	Patients with metastatic ccRCC treated in the second- or third-line setting, with no prior immune checkpoint inhibitor therapy.	a phase III, open-label, multicenter, randomized, noninferiority trial.	Nivolumab monotherapy, either subcutaneously (1200 mg every 4 weeks with rHuPH20) or intravenously (3 mg/kg every 2 weeks), based on dosing guidelines.	248/247
3475A-D77	Pembrolizumab	Participants with newly diagnosed stage IV squamous or nonsquamous NSCLC, without tumor-activating EGFR mutations or ALK/ROS1 gene rearrangements		Pembrolizumab 790 mg subcutaneously (q6w) with rHuPH20, or 400 mg intravenously (q6w), both in combination with platinum-doublet chemotherapy.	251/126
IMscin001 Part 2	Atezolizumab	Patients with documented locally advanced or metastatic NSCLC (stage IIIB not eligible for chemoradiotherapy to stage IV), cancer immunotherapy-naïve, and with progression after first-line platinum-based therapy.		Atezolizumab 1875 mg subcutaneously (q3w) with rHuPH20, or 1200 mg intravenously (q3w)	247/124

### 3.2 Quality assessment of included studies

**Figure 2:**
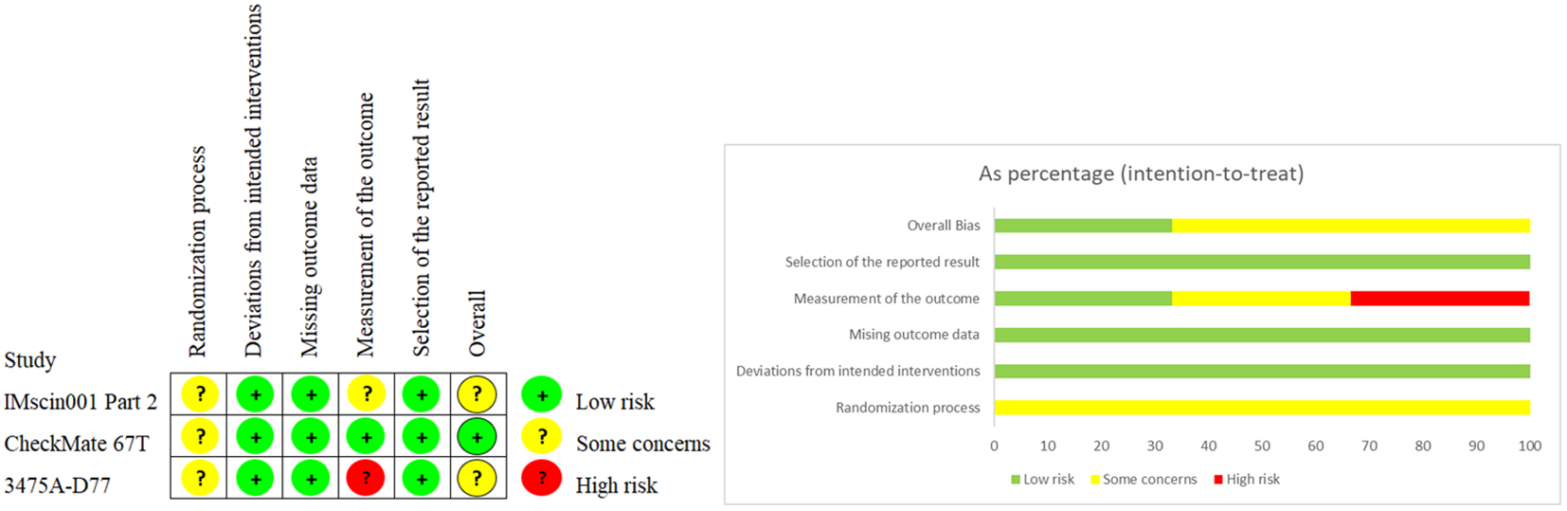
Cochrane risk of bias assessment results for included trials.

The results of the Cochrane bias risk assessment tool showed that all the included studies were of high quality, as shown in **Figure 2**.

### 3.3 Pharmacokinetics

Due to the lack of comparability in pharmacokinetic outcomes across the three trials, they could not be included in the meta-analysis. A systematic review was conducted, as detailed in **Table 2**. The pharmacokinetic outcomes included maximum concentration (C_max_), trough concentration (C_trough_), and minimum concentration (C_min_). C_trough_ was defined as the serum concentration measured immediately before the next scheduled dose at steady state, and C_min_ represented the lowest concentration observed during a specific period. The area under the concentration–time curve (AUC) reflects the total systemic exposure to the drug. The results indicate that, in terms of pharmacokinetics, the s.c. PD-1/PD-L1 inhibitors in solid tumor therapy show similar outcomes to the i.v. administration. In the CheckMate 67T study, compared to the i.v. group, the s.c. administration group exhibited nearly double the time-weighted average serum drug concentration (Cavgd28) and the minimum steady-state serum drug concentration (Cminss) during the first 28 days.

**Table 2:** Systematic review of pharmacokinetic outcomes.

Study	Drug	Time	Administration route	Indicator	Geometric mean	Geometric mean ratio
IMscin001 Part 2	Atezolizumab	Cycle 2 Day 1 (Pre-dose) after single dose	S.C.	C_trough_	89 μg/ml	1.05 (90%CI 0.88-1.24)
			I.V.	C_trough_	85 μg/ml	
		Cycle 1 Day 1–Day 21 after single dose	S.C.	AUC_0–21 d_	2907 μg•day/mL	0.87 (90%CI 0.83-0.92)
			I.V.	AUC_0–21 d_	3328 μg•day/mL	
		Cycle 2 Day 1 (Pre-dose, model-predicted) after multiple doses	S.C.	C_trough_	97 μg/ml	N/A
			I.V.	C_trough_	89 μg/ml	
		Steady state (model-predicted) after multiple doses	S.C.	C_trough ss_	205 μg/ml	N/A
			I.V.	C_trough ss_	179 μg/ml	
		Steady state(model-predicted) after multiple doses	S.C.	AUC_ss_	6163μg•day/mL	N/A
			I.V.	AUC_ss_	6107μg•day/mL	
CheckMate 67T	Nivolumab	averaged over the first 28 days	S.C.	C_avgd28_	77.373 μg/ml	2.098 (90%CI 2.001-2.200)
			I.V.	C_avgd28_	36.875 μg/ml	
		steady state	S.C.	C_min ss_	122.227 μg/ml	1.774 (90%CI 1.633-1.927 μg/ml)
			I.V.	C_min ss_	68.901 μg/ml	
3475A-D77	Pembrolizumab	Cycle 1	S.C.	AUC_0–6 weeks_	1633.24 mg•day/ml	1.14 (96%CI 1.06-1.22)
			I.V.	AUC_0–6 weeks_	1437.58 mg•day/ml	
		Cycle 3 (model-predicted)	S.C.	C_trough_	39.23 μg/ml	1.67 (96%CI 1.52-1.84)
			I.V.	C_trough_	23.49 μg/ml	
		Cycle 1 (model-predicted)	S.C.	C_trough_	19.36 μg/ml	N/A
			I.V.	C_trough_	12.26 μg/ml	
			S.C.	C_max_	64.88 μg/ml	N/A
			I.V.	C_max_	129.7 μg/ml	
		Cycle 3 (model-predicted)	S.C.	C_max_	98.96 μg/ml	N/A
			I.V.	C_max_	148 μg/ml	
			S.C.	AUC_0–6 weeks_	2798 mg•day/ml	N/A
			I.V.	AUC_0–6 weeks_	2122 mg•day/ml	

C_trough_, trough concentration; C_max_, maximum concentration; C_min_, minimum concentration; AUC, area under the concentration–time curve; S.C., subcutaneous; I.V., intravenous.

### 3.4 Survival outcomes

**Figure 3:**
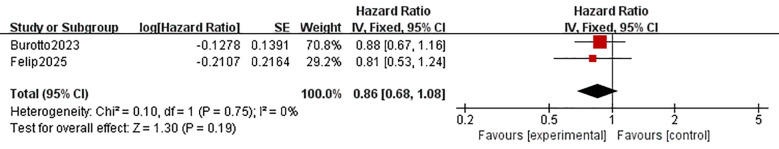
Comparison of OS between S.C. and I.V. PD-1/PD-L1 inhibitor treatment in solid tumor. OS, overall survival; S.C., subcutaneous; I.V., intravenous.

IMscin001 Part and 3475A-D77 studies reported the effect of s.c. PD-1/PD-L1 inhibitors on OS in patients with solid tumors. The results of **Figure 3** show that there was no significant difference in OS between patients in the s.c. group and the i.v. group [HR = 0.86;95% CI(0.68,1.08);P=0.19]. No significant heterogeneity was found among the trials (Chi^2^ = 0.10; df = 1 [p= 0.75]; I^2^ = 0%).

**Figure 4:**
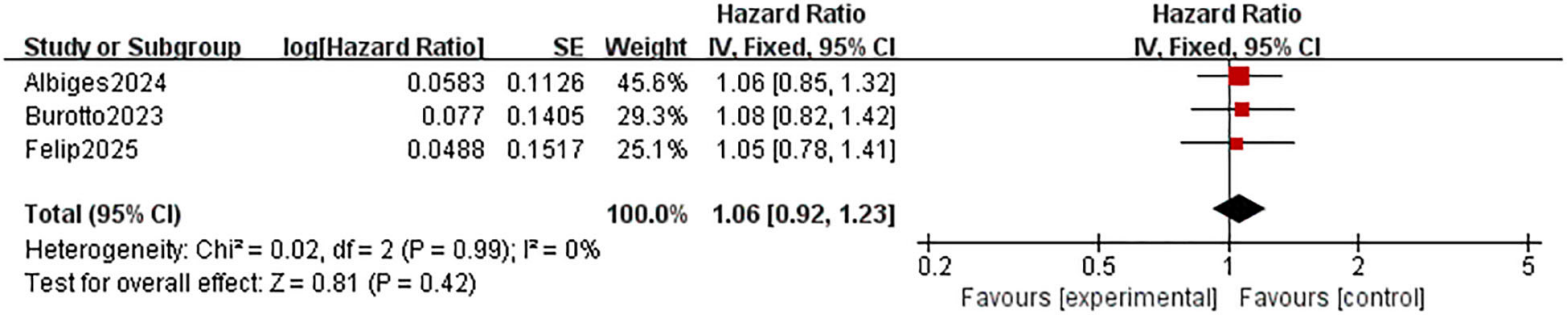
Comparison of PFS between S.C. and I.V. PD-1/PD-L1 inhibitor. Treatment in Solid Tumor. PFS, progression-free survival; S.C., subcutaneous; I.V., intravenous.

Three trials have reported the effect of s.c. PD-1/PD-L1 inhibitor treatment on PFS in patients with solid tumors. The results in **Figure 4** show that there was no significant difference in PFS between patients in the s.c. group and the i.v. group [HR = 1.06;95% CI(0.92,1.23);P=0.42]. No significant heterogeneity was found among the trials [Chi^2^ = 0.02; df = 2 (p= 0.99); I^2^ = 0%]. Sensitivity analysis by removing study by study found that no study affected the overall effect of PFS.

**Figure 5:**
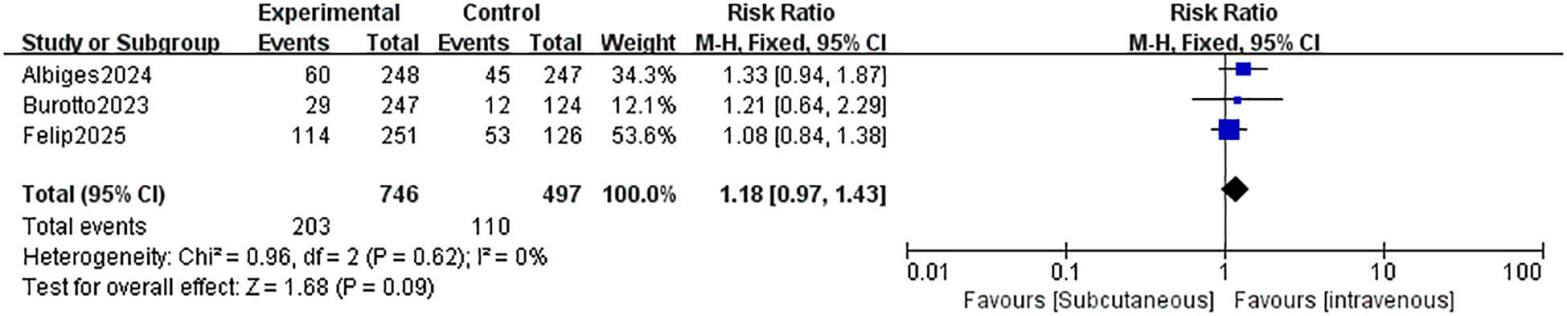
Comparison of ORR between S.C. and I.V. PD-1/PD-L1 inhibitor. Treatment in Solid Tumor. ORR, objective response rate; S.C., subcutaneous; I.V., intravenous.

All three trials reported the effect of receiving s.c. PD-1/PD-L1 inhibitor therapy on ORR in patients with solid tumors. The results in **Figure 5** show that there was no significant difference in ORR between the s.c. group and the i.v. group [RR = 1.18;95% CI(0.97,1.43);P=0.09]. No significant heterogeneity was found among the trials (Chi^2^ = 0.96; df = 2 [p= 0.62]; I^2^ = 0%). Sensitivity analysis by removing study by study found that no study affected the overall effect of ORR.

### 3.5 Safety

**Figure 6:**
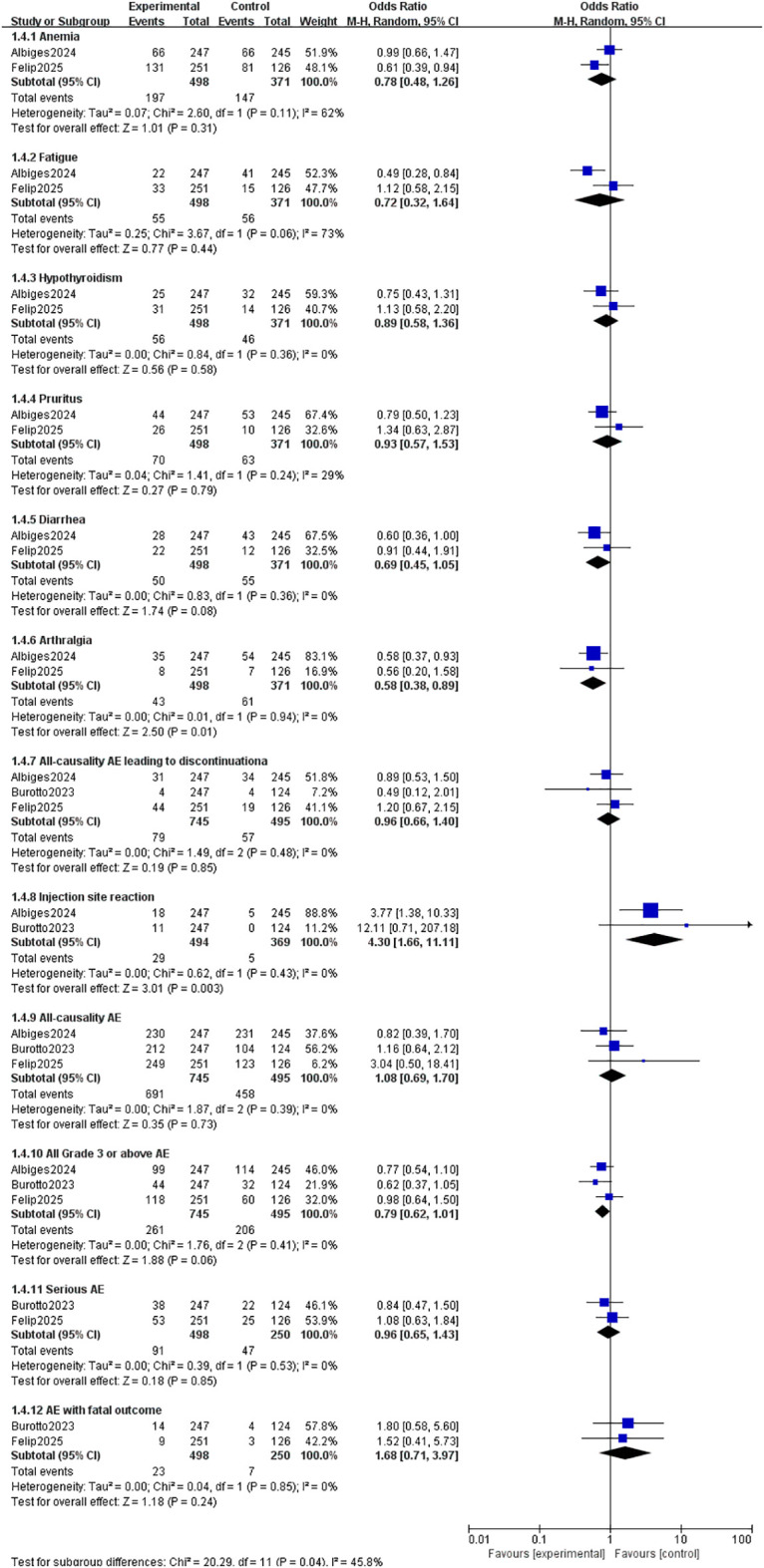
Comparison of AE between S.C. and I.V. PD-1/PD-L1 inhibitor. Treatment in Solid Tumor. AE, adverse events; S.C., subcutaneous; I.V., intravenous.

A meta-analysis of common adverse events was conducted. The results presented in **Figure 6** show that, compared to the i.v. group, the s.c. group exhibited no statistically significant differences in the incidence of Anemia [OR = 0.78; 95% CI (0.48, 1.26); P = 0.31], Fatigue [OR = 0.72; 95% CI (0.32, 1.64); P = 0.44], Hypothyroidism [OR = 0.89; 95% CI (0.58, 1.36); P = 0.58], Pruritus [OR = 0.93; 95% CI (0.57, 1.53); P = 0.79], Diarrhea [OR = 0.69; 95% CI (0.45, 1.05); P = 0.08], All-causality AE leading to discontinuationa [OR = 0.96; 95% CI (0.66, 1.40); P = 0.85], All-causality AE [OR = 1.08; 95% CI (0.69, 1.70); P = 0.73], All Grade 3 or above AE [OR = 0.79; 95% CI (0.62, 1.01); P = 0.06], Serious AE [OR = 0.96; 95% CI (0.65, 1.43); P = 0.85], and AE with fatal outcome [OR = 1.68; 95% CI (0.71, 3.97); P = 0.24]. However, Arthralgia was more common with s.c. administration [OR = 0.58; 95% CI (0.38, 0.89); P = 0.01], whereas i.v. administration was associated with a higher incidence of injection site reactions [OR = 4.30; 95% CI (1.66, 11.11); P = 0.003]. After Bonferroni correction, the corrected P value was 0.004. Thus, the results were statistically different only for the subgroup of injection sites (P = 0.003< 0.004).

## 4 Discussion

The transition of PD-1/PD-L1 inhibitors from i.v. to s.c. administration is gradually being realized. In August 2023, Atezolizumab received approval from the UK Medicines and Healthcare products Regulatory Agency for s.c. administration (11). This approval marks Atezolizumab as the first PD-1/PD-L1 inhibitor to switch from i.v. to s.c. administration, applicable to all approved indications (5). Although this administration route has not yet been approved by the FDA or the EMA, the introduction of s.c. formulations highlights the growing clinical acceptance of alternative administration routes and may represent a significant future direction in the development of immunotherapeutic agents.

Therefore, our study included three pivotal clinical trials: IMscin001 Part 2, CheckMate 67T, and 3475A-D77. These trials evaluated three PD-1/PD-L1 inhibitors: Atezolizumab, Nivolumab, and Pembrolizumab. The aim is to systematically compare the pharmacokinetics, efficacy, and safety of PD-1/PD-L1 inhibitors administered via s.c. and i.v. in the treatment of solid tumors. Our study also explored the advantages and limitations of both administrations, aiming to provide evidence-based support for clinical practice.

All three clinical trials shared pharmacokinetic non-inferiority as the primary endpoint, utilizing subcutaneously administered PD-1/PD-L1 inhibitors formulated with recombinant human hyaluronidase (rHuPH20). Several studies have demonstrated that rHuPH20-based s.c. formulations in cancer immunotherapy exhibit comparable benefit-risk profiles to traditional i.v. formulations, thereby ensuring their efficacy and safety (12–14). In the three trials, the single-dose administration of the s.c. formulation was higher than that of the i.v. formulation, aiming to achieve comparable target saturation and to reach the exposure levels required for therapeutic efficacy. The researchers compared serum drug concentration after multiple doses to steady state. The results indicated that the s.c. group was non-inferior to the i.v. group in terms of pharmacokinetic parameters, suggesting that it has the potential to maintain stable efficacy in long-term treatment. However, the geometric mean ratios of these three studies were very different, for example, 2.098 for Cavgd_28_ in CheckMate 67T, 1.14 for AUC_0–6 weeks_ in 3475A-D77, and 0.87 for AUC_0-21d_ in IMscin001. There were some differences between PD-1/PD-L1 inhibitors and rHuPH 20 configured concentrations in the 3 studies. In CheckMate 67T, Nivolumab s.c. was administered subcutaneously at a fixed dose of 1200 mg every 4 weeks in combination with rHuPH20 20,000 units(U). Atezolizumab s.c. (15 ml) in IMscin001 was administered subcutaneously in a co-formulation of 1875 mg and 30,000 U rHuPH20. In 3475A-D77, Pembrolizumab s.c. 790 mg was administered subcutaneously in a solution specification of 165 mg/mL and rHuPH20–2000 U/mL in a total injection volume of approximately 4.8 mL. This difference in configuration concentration may account for the large difference in geometric mean ratios.

In terms of immunogenicity, all three clinical trials revealed that the incidence of treatment-induced anti-drug antibodies (ADA) was higher in the s.c. group compared to the i.v. group. The incidence rates of ADA in the s.c. groups reported by CheckMate 67T and IMscin001 were 24% and 20.6% respectively, significantly higher than those in the corresponding i.v. groups (7% and 14.3%); although the overall ADA incidence rate was lower in 3475A-D77, it also showed a similar trend (1.40% in the s.c. group vs. 0.9% in the i.v. group). This phenomenon has always been one of the main problems with s.c. administration of biological agents. This may be attributed to the enhanced antigen presentation by highly active antigen-presenting cells, such as dendritic cells, in the skin during s.c. administration, which could lead to an increased immune response (15). However, all three clinical trials indicated that the differences in ADA between the groups did not have a clinically significant negative impact on the pharmacokinetics, clinical efficacy, or safety of the drug. This is consistent with the conclusions of two large head-to-head studies comparing s.c. and i.v. administration in the field of tumor-targeted therapy (16). ADA positivity differences among s.c. groups were associated with multiple factors, including drug formulation, patient disease status, molecular mechanism of action, dosing frequency and others (16). Current evidence shows no impact of the higher immunogenicity risk with s.c. administration on antitumor efficacy.

On the basis of ensuring pharmacokinetic non-inferiority, all three clinical trials demonstrated that the s.c. group exhibited similar efficacy to the i.v. group, as evidenced by comparable outcomes in OS, PFS, and ORR. The CheckMate 67T study further analyzed disease control rate (DCR) and time to response (TTR), the results showed that both administrations yielded similar outcomes in terms of disease control and time to onset of effect. The DCR in the s.c. group was 62.5%, and the median TTR was 3.71 months; the DCR in the i.v. group was 62.8%, and the median TTR was 3.68 months. In addition, the 3475A-D77 study analyzed data such as ORR, median PFS, and median DOR in the s.c. and i.v. groups with the representative studies of pembrolizumab i.v. for metastatic non-small cell lung cancer, namely KEYNOTE-189 (17) and KEYNOTE-407 (18). The comparative results suggested consistency, supporting the non-inferiority of the s.c. formulation in terms of efficacy. It should be noted that the follow-up duration of the three included trials did not exceed three years, and survival and efficacy outcomes were designated as secondary endpoints in the trials. Therefore, the OS data may be subject to immaturity. We believe that the conclusion of efficacy equivalence between s.c. and i.v. administration requires further confirmation with longer follow-up data, and any interpretation at the current stage should be made with caution.

Safety is one of the key considerations in the promotion of s.c. administration of PD-1/PD-L1 inhibitors. In the three studies, the safety of the s.c. and i.v. administrations were generally similar, with no unexpected safety concerns identified. It is important to note that PD-1/PD-L1 inhibitors can overstimulate T cells and release inflammatory factors (19), leading to immune-related adverse events such as joint pain. Subcutaneous injection may exacerbate this concern.

Based on the aforementioned premises, the reduction of clinical time requirements and the improvement of patient experience are the primary advantages of the s.c. administration. In three clinical trials, s.c. administration required 2 to 8 minutes, markedly shorter than the 30 to 40 minutes for i.v. administration. Previous studies have confirmed that shortening treatment time through s.c. administration can effectively reduce healthcare resource utilization and costs, thereby optimizing the long-term management model of cancer therapy (20, 21). The IMscin001 Part2 and CheckMate 67T studies both assessed patient preferences for different routes of administration, and the results showed that patients preferred s.c. administration or were neutral towards it compared to i.v. administration.

Optimization of monoclonal antibody delivery via subcutaneous formulations aligns with the broader biomedical engineering landscape for the optimization and personalization of therapies. For example, innovations in tissue engineering and controlled release for cardiovascular health have demonstrated the value of optimizing the delivery route for more effective and more compliant therapies (22). The application of these concepts and technologies in the context of cancer treatment via either engineered subcutaneous formulations or in combination with recombinant human hyaluronidase represents the fusion of immuno-oncology and precision delivery science.

Over the past decade, PD-1/PD-L1 inhibitors have represented a major breakthrough in the treatment of advanced solid tumors. PD-1/PD-L1 inhibitors have significantly altered the landscape of cancer therapy, serving as effective combination or alternatives to chemotherapy and targeted therapy, and offering new possibilities for long-term remission in various tumor types. The clinical application of PD-1/PD-L1 inhibitors has now surpassed that of anti-CTLA-4 inhibitors. Since the approval of PD-1/PD-L1 inhibitors, the instructions for use of these inhibitors have been continuously optimized in clinical practice. For example, during the COVID-19 pandemic in 2019, the administration frequency of Nivolumab was adjusted to reduce the frequency of high-dose administration, thereby effectively lowering healthcare costs and treatment duration while improving patient adherence (23). Although s.c. administration has significant advantages in shortening administration time, reducing patient discomfort, and supporting self-administration, all PD-1/PD-L1 inhibitors approved by the FDA and EMA currently still use i.v. administration. Therefore, how to expand the global application and accessibility of these increasingly important anticancer agents has emerged as an urgent issue that needs to be addressed. The subcutaneous administration of PD-1/PD-L1 inhibitors undoubtedly represents a promising area of interest. Clinical trials investigating subcutaneous formulations of Tislelizumab (CTR20253206) and Toripalimab (CTR20241554) are currently in the recruitment phase, and our study provides preliminary evidence supporting this emerging therapeutic direction.

Looking ahead, the evolution of subcutaneous immunotherapy may increasingly intersect with emerging diagnostic technologies. For example, CRISPR-microfluidic linkage may soon be able to monitor drug levels, anti-drug antibodies, or even tumor DNA in real time at the point of care (24). Such integration could transform subcutaneous administration of immunotherapies from a passive drug delivery alternative into an active part of a closed-loop personalized medicine system, where drug dosage, timing, and even formulation are dynamically adjusted based on patient pharmacokinetics and tumor response. The rHuPH20-based formulations evaluated in this meta-analysis are a first step towards this vision and establish the safety and efficacy foundation upon which such innovations can be built.

This study has certain limitations. The number of available clinical trials is limited. Subgroup analysis by tumor type or dosing regimen is not feasible. This limitation may reduce the generalizability of the findings. Future research should conduct larger-scale prospective clinical studies to further validate the real-world clinical value of s.c. PD-1/PD-L1 inhibitors.

## 5 Conclusion

Overall, our findings indicate that s.c. PD-1/PD-L1 inhibitors exhibit similar pharmacokinetics to i.v. administration in the treatment of solid tumors, with no significant differences in efficacy and safety. These results provide evidence to support the broader clinical application of s.c. administration in indications currently approved for i.v. PD-1/PD-L1 inhibitors, supporting s.c. administration as a novel and complementary alternative to the conventional i.v. route.

## Data Availability

The original contributions presented in the study are included in the article/**Supplementary Material**. Further inquiries can be directed to the corresponding author.
