# Supplementary material for: Efficacy and safety of subcutaneous versus intravenous administration of PD-1/PD-L1 inhibitors in the treatment of solid tumors: a systematic review and meta-analysis
Source: Front Oncol. 2026 Apr 27;16:1748414. doi: 10.3389/fonc.2026.1748414 (PMC13158120; doi:10.3389/fonc.2026.1748414)
Supplement: Supplementary file 1 [file Table1.docx]

Supplementary Material

**eMethods**

**PubMed <** **Database established until October 10, 2025> Search Strategy (71)**

#1 "pembrolizumab" [Supplementary Concept]

#2 pembrolizumab[Title/Abstract] OR MK-3475[Title/Abstract] OR Keytruda[Title/Abstract] OR lambrolizumab[Title/Abstract] OR SCH-900475[Title/Abstract]

#3 "nivolumab"[Mesh]

#4 nivolumab[Title/Abstract] OR MDX-1106[Title/Abstract] OR MDX1106[Title/Abstract] OR MDX 1106[Title/Abstract] OR Opdivo[Title/Abstract] OR BMS-936558[Title/Abstract] OR BMS936558[Title/Abstract] OR BMS 936558[Title/Abstract] OR ONO-4538[Title/Abstract] OR ONO4538[Title/Abstract] OR ONO 4538[Title/Abstract]

#5 "atezolizumab" [Supplementary Concept]

#6 atezolizumab[Title/Abstract] OR immunoglobulin G1, anti-human CD antigen CD274 human monoclonal MDPL3280a heavy chain, disulfide with human monoclonal MDPL3280a kappa-chain, dimer[Title/Abstract] OR anti-PDL1[Title/Abstract] OR MPDL3280A[Title/Abstract] OR MPDL-3280A[Title/Abstract] OR Tecentriq[Title/Abstract] OR RG7446[Title/Abstract] OR RG-7446[Title/Abstract]

#7 "durvalumab" [Supplementary Concept]

#8 durvalumab[Title/Abstract] OR MEDI4736[Title/Abstract] OR MEDI-4736[Title/Abstract] OR Imfinzi[Title/Abstract]

#9 "avelumab" [Supplementary Concept]

#10 avelumab[Title/Abstract] OR MSB0010718C[Title/Abstract] OR MSB-0010718C[Title/Abstract] OR bavencio[Title/Abstract] OR MSB-0010682[Title/Abstract] OR MSB0010682[Title/Abstract]

#11 "toripalimab" [Supplementary Concept]

#12 toripalimab[Title/Abstract] OR toripalimab-tpzi[Title/Abstract]

#13 "sintilimab" [Supplementary Concept]

#14 sintilimab[Title/Abstract] OR IBI 308[Title/Abstract] OR IBI308[Title/Abstract] OR IBI-308[Title/Abstract]

#15 "camrelizumab" [Supplementary Concept]

#16 camrelizumab[Title/Abstract] OR SHR-1210[Title/Abstract] OR SHR 1210[Title/Abstract] OR carrelizumab[Title/Abstract]

#17 "Immune Checkpoint Inhibitors"[Mesh]

#18 Immune Checkpoint Inhibitors[Title/Abstract] OR Checkpoint Inhibitors, Immune[Title/Abstract] OR Immune Checkpoint Blockers[Title/Abstract] OR Checkpoint Blockers, Immune[Title/Abstract] OR Immune Checkpoint Inhibitor[Title/Abstract] OR Checkpoint Inhibitor, Immune[Title/Abstract] OR PD-1 Inhibitors[Title/Abstract] OR PD 1 Inhibitors[Title/Abstract] OR Programmed Cell Death Protein 1 Inhibitor[Title/Abstract] OR Programmed Cell Death Protein 1 Inhibitors[Title/Abstract] OR PD-1 Inhibitor[Title/Abstract] OR Inhibitor, PD-1[Title/Abstract] OR PD 1 Inhibitor[Title/Abstract] OR Immune Checkpoint Blockade[Title/Abstract] OR Checkpoint Blockade, Immune[Title/Abstract] OR Immune Checkpoint Inhibition[Title/Abstract] OR Checkpoint Inhibition, Immune[Title/Abstract] OR PD-L1 Inhibitors[Title/Abstract] OR PD L1 Inhibitors[Title/Abstract] OR Programmed Death-Ligand 1 Inhibitors[Title/Abstract] OR Programmed Death Ligand 1 Inhibitors[Title/Abstract] OR PD-L1 Inhibitor[Title/Abstract] OR PD L1 Inhibitor[Title/Abstract] OR PD-1-PD-L1 Blockade[Title/Abstract] OR Blockade, PD-1-PD-L1[Title/Abstract] OR PD 1 PD L1 Blockade[Title/Abstract]

#19 #1 OR #2 OR #3 OR #4 OR #5 OR #6 OR #7 OR #8 OR #9 OR #10 OR #11 OR #12 OR #13 OR #14 OR #15 OR #16 OR #17 OR #18

#20 "Injections, Intravenous"[Mesh] OR Intravenous[Title/Abstract]

#21 "Injections, Subcutaneous"[Mesh] OR Subcutaneous[Title/Abstract]

#22 #19 AND #20 AND #21

**Embase < Database established until October 10, 2025> Search Strategy (74)**

#1 'pembrolizumab'/exp

#2 'bcd 201':ab,kw,ti OR 'bcd201':ab,kw,ti OR 'keytruda':ab,kw,ti OR 'lambrolizumab':ab,kw,ti OR 'mk 3475':ab,kw,ti OR 'mk3475':ab,kw,ti OR 'pbp 2102':ab,kw,ti OR 'pbp2102':ab,kw,ti OR 'sch 900475':ab,kw,ti OR 'sch900475':ab,kw,ti OR 'xtrudane':ab,kw,ti OR 'pembrolizumab':ab,kw,ti

#3 'nivolumab'/exp

#4 'ba 1104':ab,kw,ti OR 'ba1104':ab,kw,ti OR 'bms 936558':ab,kw,ti OR 'bms936558':ab,kw,ti OR 'cmab 819':ab,kw,ti OR 'cmab819':ab,kw,ti OR 'ly 01015':ab,kw,ti OR 'ly01015':ab,kw,ti OR 'mdx 1106':ab,kw,ti OR 'mdx1106':ab,kw,ti OR 'ono 4538':ab,kw,ti OR 'ono4538':ab,kw,ti OR 'opdivo':ab,kw,ti OR 'pbp 2101':ab,kw,ti OR 'pbp2101':ab,kw,ti OR 'xdivane':ab,kw,ti OR 'nivolumab':ab,kw,ti

#5 'atezolizumab'/exp

#6 'monoclonal antibody mpdl 3280a':ab,kw,ti OR 'monoclonal antibody mpdl3280a':ab,kw,ti OR 'mpdl 3280a':ab,kw,ti OR 'mpdl3280a':ab,kw,ti OR 'rg 7446':ab,kw,ti OR 'rg7446':ab,kw,ti OR 'ro 5541267':ab,kw,ti OR 'ro5541267':ab,kw,ti OR 'tecentriq':ab,kw,ti OR 'tecntriq':ab,kw,ti OR 'atezolizumab':ab,kw,ti

#7 'durvalumab'/exp

#8 'imfinzi':ab,kw,ti OR 'medi 4736':ab,kw,ti OR 'medi4736':ab,kw,ti OR 'durvalumab':ab,kw,ti

#9 'avelumab'/exp

#10 'bavencio':ab,kw,ti OR 'msb 0010682':ab,kw,ti OR 'msb 0010718c':ab,kw,ti OR 'msb 10682':ab,kw,ti OR 'msb 10718c':ab,kw,ti OR 'msb0010682':ab,kw,ti OR 'msb0010718c':ab,kw,ti OR 'msb10682':ab,kw,ti OR 'msb10718c':ab,kw,ti OR 'pf 06834635':ab,kw,ti OR 'pf 6834635':ab,kw,ti OR 'pf06834635':ab,kw,ti OR 'pf6834635':ab,kw,ti OR 'avelumab':ab,kw,ti

#11 'toripalimab'/exp

#12 'chs 007':ab,kw,ti OR 'chs007':ab,kw,ti OR 'js 001':ab,kw,ti OR 'js001':ab,kw,ti OR 'loqtorzi':ab,kw,ti OR 'tab 001':ab,kw,ti OR 'tab001':ab,kw,ti OR 'teripalimab':ab,kw,ti OR 'teriprizumab':ab,kw,ti OR 'toripalimab tpzi':ab,kw,ti OR 'toripalimab-tpzi':ab,kw,ti OR 'treipril':ab,kw,ti OR 'treprizumab':ab,kw,ti OR 'tripleitriumab':ab,kw,ti OR 'triprizumab':ab,kw,ti OR 'tuoyi':ab,kw,ti OR 'toripalimab':ab,kw,ti

#13 'sintilimab'/exp

#14 'ibi 308':ab,kw,ti OR 'ibi308':ab,kw,ti OR 'tyvyt':ab,kw,ti OR 'sintilimab':ab,kw,ti

#15 'camrelizumab'/exp

#16 'airuika':ab,kw,ti OR 'hr 301210':ab,kw,ti OR 'hr301210':ab,kw,ti OR 'incshr 1210':ab,kw,ti OR 'incshr1210':ab,kw,ti OR 'shr 1210':ab,kw,ti OR 'shr1210':ab,kw,ti OR 'camrelizumab':ab,kw,ti

#17 'immune checkpoint inhibitor'/exp

#18 'immune checkpoint blocker':ab,kw,ti OR 'immune checkpoint inhibitors':ab,kw,ti OR 'immune checkpoint inhibitor':ab,kw,ti

#19 #1 OR #2 OR #3 OR #4 OR #5 OR #6 OR #7 OR #8 OR #9 OR #10 OR #11 OR #12 OR #13 OR #14 OR #15 OR #16 OR #17 OR #18

#20 'intravenous drug administration'/exp

#21 'administration, intravenous':ab,kw,ti OR 'drug administration, intravenous':ab,kw,ti OR 'infusion, intravenous':ab,kw,ti OR 'infusions, intravenous':ab,kw,ti OR 'injection, intravenous':ab,kw,ti OR 'injections, intravenous':ab,kw,ti OR 'intravenous administration':ab,kw,ti OR 'intravenous bolus administration':ab,kw,ti OR 'intravenous bolus drug administration':ab,kw,ti OR 'intravenous bolus injection':ab,kw,ti OR 'intravenous dose':ab,kw,ti OR 'intravenous drip':ab,kw,ti OR 'intravenous drip administration':ab,kw,ti OR 'intravenous drip infusion':ab,kw,ti OR 'intravenous drip injection':ab,kw,ti OR 'intravenous fluid administration':ab,kw,ti OR 'intravenous fluid therapy':ab,kw,ti OR 'intravenous infusion':ab,kw,ti OR 'intravenous infusions':ab,kw,ti OR 'intravenous injection':ab,kw,ti OR 'intravenous injections':ab,kw,ti OR 'intravenous medication':ab,kw,ti OR 'intravenous therapy':ab,kw,ti OR 'intravenous transfusion':ab,kw,ti OR 'iv administration':ab,kw,ti OR 'iv drug administration':ab,kw,ti OR 'iv drug delivery':ab,kw,ti OR 'iv drug injection':ab,kw,ti OR 'iv drug therapy':ab,kw,ti OR 'iv fluid administration':ab,kw,ti OR 'iv infusion':ab,kw,ti OR 'iv injection':ab,kw,ti OR 'iv medication':ab,kw,ti OR 'iv transfusion':ab,kw,ti OR 'vein infusion':ab,kw,ti OR 'vein injection':ab,kw,ti OR 'venous drip':ab,kw,ti OR 'venous infusion':ab,kw,ti OR 'venous injection':ab,kw,ti OR 'venous transfusion':ab,kw,ti OR 'intravenous drug administration':ab,kw,ti

#22 #20 OR #21

#23 'subcutaneous drug administration'/exp

#24 'drug administration, subcutaneous':ab,kw,ti OR 'infusion, subcutaneous':ab,kw,ti OR 'infusions, subcutaneous':ab,kw,ti OR 'injections, subcutaneous':ab,kw,ti OR 'subcutaneous administration':ab,kw,ti OR 'subcutaneous application':ab,kw,ti OR 'subcutaneous dosage':ab,kw,ti OR 'subcutaneous dose':ab,kw,ti OR 'subcutaneous drug injection':ab,kw,ti OR 'subcutaneous infusion':ab,kw,ti OR 'subcutaneous infusions':ab,kw,ti OR 'subcutaneous injection':ab,kw,ti OR 'subcutaneous injections':ab,kw,ti OR 'subcutaneous drug administration':ab,kw,ti

#25 #23 OR #24

#26 #19 AND #22 AND #25

**Cochrane library < Updated from 2015-01-15 to 2025-01-15> Search Strategy (24)**

#1 MeSH descriptor: [Nivolumab] explode all trees

#2 (“MDX1106” OR “MDX-1106” OR “MDX 1106” OR “BMS-936558” OR “BMS936558” OR “BMS 936558” OR “ONO 4538” OR “ONO4538” OR “ONO-4538” OR “Nivolumab” OR “Opdivo”) :ti,ab,kw

#3 (“Pembrolizumab” OR “MK-3475” OR “Keytruda” OR “lambrolizumab” OR “SCH-900475”) :ti,ab,kw

#4 (“Atezolizumab” OR “immunoglobulin G1, anti-”human CD antigen CD274” “human monoclonal MDPL3280a heavy chain”, disulfide with human monoclonal MDPL3280a kappa-chain, dimer” OR “anti-PDL1” OR “MPDL3280A” OR “MPDL-3280A” OR “Tecentriq” OR “RG7446” OR “RG-7446”) :ti,ab,kw

#5 (“Durvalumab” OR “MEDI4736” OR “MEDI-4736” OR “Imfinzi”) :ti,ab,kw

#6 (“Avelumab” OR “MSB0010718C” OR “MSB-0010718C” OR “bavencio” OR “MSB-0010682” OR “MSB0010682”) :ti,ab,kw

#7 (“Toripalimab” OR “toripalimab-tpzi”) :ti,ab,kw

#8 (“Sintilimab” OR “IBI 308” OR “IBI308” OR “IBI-308”) :ti,ab,kw

#9 MeSH descriptor: [Immune Checkpoint Inhibitors] explode all trees

#10 ("Immune Checkpoint Inhibitors" OR "Checkpoint Inhibitors, Immune" OR "Immune Checkpoint Blockers" OR "Checkpoint Blockers, Immune" OR "Immune Checkpoint Inhibitor" OR "Checkpoint Inhibitor, Immune" OR "PD-1 Inhibitors" OR "PD 1 Inhibitors" OR "Programmed Cell Death Protein 1 Inhibitor" OR "Programmed Cell Death Protein 1 Inhibitors" OR "PD-1 Inhibitor" OR "Inhibitor, PD-1" OR "PD 1 Inhibitor" OR "Immune Checkpoint Blockade" OR "Checkpoint Blockade, Immune" OR "Immune Checkpoint Inhibition" OR "Checkpoint Inhibition, Immune" OR "PD-L1 Inhibitors" OR "PD L1 Inhibitors" OR "Programmed Death-Ligand 1 Inhibitors" OR "Programmed Death Ligand 1 Inhibitors" OR "PD-L1 Inhibitor" OR "PD L1 Inhibitor" OR "PD-1-PD-L1 Blockade" OR "Blockade, PD-1-PD-L1" OR "PD 1 PD L1 Blockade"):ti,ab,kw

#11 #1 OR #2 OR #3 OR #4 OR #5 OR #6 OR #7 OR #8 OR #9 OR #10

#12 MeSH descriptor: [Administration, Intravenous] explode all trees

#13 (“Intravenous Administrations” OR “ Administrations, Intravenous” OR “ Intravenous Administration”):ti,ab,kw

#14 #12 OR #13

#15 MeSH descriptor: [Injections, Subcutaneous] explode all trees

#16 (“Subcutaneous Injection”:ti,ab,kw OR “ Injection, Subcutaneous”:ti,ab,kw OR “ Subcutaneous Injections”):ti,ab,kw

#17 #15 OR #16

#18 #11 AND #14 AND #17
